# Enhancing fieldwork readiness in occupational therapy students with generative AI

**DOI:** 10.3389/fmed.2024.1485325

**Published:** 2024-10-16

**Authors:** Tara Mansour, John Wong

**Affiliations:** MGH Institute of Health Professions, Boston, MA, United States

**Keywords:** generative AI, evidence-based practice, occupational therapy, fieldwork, clinical education, intervention planning

## Abstract

The rapid integration of artificial intelligence (AI) into health professions education is revolutionizing traditional teaching methodologies and enhancing learning experiences. This study explores the use of generative AI to aid occupational therapy (OT) students in intervention planning. OT students often lack the background knowledge to generate a wide variety of interventions, spending excessive time on idea generation rather than clinical reasoning, practice skills, and patient care. AI can enhance creative ideation but students must still adhere to evidence-based practice, patient safety, and privacy standards. Students used ChatGPT v. 3.5 in a lecture and assignment to integrate generative AI into intervention planning. Students analyzed a case study, generated ideas with ChatGPT, selected interventions that aligned with the client’s needs, and provided a rationale. They conducted evidence-based searches and wrote an analysis on how the research influenced their decisions. The results demonstrate generative AI’s potential as a valuable tool for OT students, enhancing their comfort with AI and understanding of ethical and safety considerations. Qualitative feedback highlighted AI’s role in boosting efficiency and creativity in intervention planning, with most students expressing strong intent to use ChatGPT in clinical practice due to its ability to reduce cognitive load and generate innovative ideas. These findings suggest that integrating generative AI into the OT curriculum could enhance intervention planning and improve clinical readiness.

## Introduction

### Problem description

Navigating the challenges of creative intervention planning can pose a significant challenge for occupational therapy (OT) students during their full-time clinical placements, otherwise known as Level II Fieldwork. Many OT students have expressed a sense of lacking concrete interventional knowledge, resulting in feelings of incompetence and uncertainty regarding their ability to deliver effective interventions, and a desire for more examples to draw from in clinical practice (1, 2). Developing intervention planning skills in Level II fieldwork prepares students for independent practice and a successful transition into the OT role (2).

Struggling with intervention planning can significantly increase the cognitive load for OT students during Level II fieldwork. The effort required to generate and refine intervention strategies may divert attention and mental resources away from other crucial aspects of client care, such as clinical reasoning, complex medical management of lines and tubes and therapeutic rapport building (3). This heightened cognitive load may hinder students’ overall performance, potentially leading to feelings of frustration and decreased confidence in their clinical abilities (4). Sewell et al. (5) explored the application of cognitive load theory in healthcare education and training. The review highlights the impact of cognitive load on performance, emphasizing the challenges faced by trainees, particularly novices dealing with complex tasks as well as the implications of high cognitive load on learning, performance, and well-being of healthcare professionals. Posciask et al. (4) emphasize the importance of structuring instruction to reduce cognitive load and improve learning outcomes.

### Available knowledge

AI has revolutionized various fields and has shown promise in various applications within the health professions (6). Capable of using algorithms to create new content and ideas, generative AI is increasingly integral to various aspects of medicine, offering significant improvements in diagnostics, clinical decision-making, and patient management. In the field of dermatology, AI is employed to enhance the diagnostic accuracy of skin cancer, rivaling even experienced dermatologists (7). AI’s implementation extends into perioperative medicine, where it optimizes anesthesiology practices by predicting patient-specific risks and improving the precision of medical interventions underscoring AI’s potential to augment human expertise in visual diagnostic tasks (8). AI’s utility in evidence-based medicine is profound, especially when integrated with decision support systems to validate and explain medical decisions which ensures that AI’s output is not only accurate but also understandable to clinicians, which is essential for trust and ethical integration into clinical practice (9). AI’s broad applicability across various medical specialties highlights its transformative impact on healthcare, providing both operational efficiencies and enhanced patient care outcomes (7, 9).

Building on the growing role of AI in medicine, its application in health professions education holds the potential to transform how future clinicians are trained. By integrating AI into educational environments, it can complement human capabilities, promote critical thinking, and improve educational outcomes (10, 11). The integration of AI in healthcare education, particularly using tools like generative AI for intervention planning, is an emerging area with limited existing research. To the authors’ knowledge, there is limited research specifically exploring the use of AI to aid OT students in creating treatment plans. AI can help occupational therapy students generate intervention ideas that are personalized and efficient. Qu et al. (10) report that using AI tools such as ChatGPT can decrease cognitive load by automating routine tasks, allowing students to conserve mental energy for higher-order cognitive functions such as clinical reasoning. By offloading repetitive tasks to generative AI, students can focus more on critical thinking, problem-solving, and decision-making processes essential for clinical reasoning. This reduction in cognitive load can empower students to engage more deeply with complex clinical scenarios and enhance their ability to apply theoretical knowledge to practical situations (10).

Despite the growing potential of AI in education, there are several limitations that need to be considered. One key concern is the risk of over-reliance on AI, which can impede the development of critical thinking, decision-making, and analytical reasoning abilities by encouraging the uncritical acceptance of AI-generated information (12). While AI can assist with automating repetitive tasks, excessive reliance on these tools can diminish students’ ability to solve complex problems independently, as over-dependence may reduce their cognitive engagement and critical thinking (13).

### Rationale

The rationale for integrating generative AI into OT education is grounded in the need for innovative educational tools that can enhance learning and application in clinical practice. The authors hypothesize that generative AI, as a cognitive aid, can facilitate the generation of diverse intervention ideas while simultaneously reducing the cognitive load associated with this novel task. This reduction is expected to allow students to focus more on refining their clinical reasoning and applied practice skills, rather than on the initial generation of ideas. In addition, by providing a broad array of intervention options, generative AI may also help students to overcome the limitations of their current experience or background knowledge, promoting a more dynamic and creative approach to clinical problem-solving. The use of generative AI is anticipated to act as a bridge between theoretical knowledge and practical application, enhancing the quality of patient care through more informed and innovative intervention strategies.

### Specific aims

The specific aims of the study include:

1. Demonstrate the potential of generative AI to act as an effective educational tool by providing data on changes in students’ confidence and ability to generate intervention ideas both before and after using ChatGPT.
2. Explore the impact of generative AI on the educational experience of OT students in terms of engagement, learning efficiency, and satisfaction.
3. Identify and discuss the benefits and limitations of using AI technologies like ChatGPT in OT education, particularly focusing on its role in supporting evidence-based practice and maintaining patient safety and confidentiality.
4. Propose recommendations for integrating AI tools into OT curricula and suggest areas for further research based on the findings of this exploratory study.

## Methods

### Context

This study was conducted over a two-week period within a fieldwork seminar course taken by entry-level occupational therapy doctoral (EL-OTD) students during their final semester of didactic work before transitioning to full-time clinical placements. This course is designed to prepare students for participation in full-time Level II fieldwork in OT practice settings. The learning objectives focus on enhancing students’ ability to deliver occupational therapy services under supervision, with an emphasis on safety, ethics, evaluation, intervention planning, and professional behaviors. The students had completed extensive coursework in evidence-based practice and clinical reasoning. However, most had limited prior experience using AI tools in their academic work. Students were informed about the study. Although completing the assignment was mandatory, participation in the pre-and post-surveys were voluntary.

### Intervention

The intervention, which included a lecture and an assignment, integrated ChatGPT v. 3.5, an AI-driven tool, into the fieldwork seminar course curriculum to assist students in generating diverse intervention strategies. ChatGPT v. 3.5 was chosen for its contextually relevant responses and accessibility to all students. During the lecture, the instructor presented a case study, conducted an intervention search using ChatGPT, and selected three options generated by the tool. One of the selected interventions was intentionally chosen because it was contraindicated - meaning it was not appropriate or safe for the specific case due to the patient’s condition. Next, students were prompted to evaluate the interventions, identify risks, and apply evidence-based reasoning to avoid contraindicated options. Students were required to perform a literature review on each of the chosen strategies to determine whether they would proceed with the intervention and why, emphasizing the importance of evidence-based practice. Following the lecture, students were required to complete an assignment designed to equip them with the knowledge and skills required to integrate generative AI technology responsibly and ethically into OT practice. Using a case study, different from the one presented in the lecture, students were required to explore the potential of ChatGPT as a valuable tool for generating intervention ideas while concurrently developing an understanding of the critical considerations surrounding patient confidentiality, ethics, safety, and evidence-based practice. Students were required to use ChatGPT to generate intervention ideas for the case study and then justify their selections by referencing evidence-based resources such as research articles, textbooks, and post-surgical protocols. Further reflection required students to use the gathered information to determine whether the ChatGPT-generated interventions should be implemented or not, and to explain their reasoning. Participants were given approximately 1 week to complete the assignment. The assignment was designed to reinforce the learning objectives and provide practical application opportunities for participants.

### Data collection

To assess the impact of ChatGPT on the students’ learning outcomes a mixed methods sequential convergent design was employed to analyze the data. This approach involved two distinct phases. First, a quantitative statistical analysis comparing data from the pre-intervention survey (*n* = 34) to that gathered in the post-intervention survey (n = 27) where the intervention was the lecture and assignment. This was followed by descriptive data collection and qualitative data analysis of responses from 27 students in the post-intervention survey (14). In this design, qualitative data were analyzed after the quantitative phase to provide deeper insights into the participants views. The rationale for this approach is that the quantitative data provides a general understanding of whether there was a statistically significant change as a result of the intervention, while the qualitative data refines and explains the statistical results by exploring participants’ experiences in more depth. The Institutional Review Board determined that this project did not meet the criteria for human subjects research because the data was not intended to generate generalized knowledge. Instead, it was designed to evaluate the effectiveness of the intervention within the context of the course, aiming to enhance the quality of the assignment.

### Measures

Based on the study’s objectives, the researchers self-developed quantitative and qualitative questions. To ensure content and construct validity, the questions were reviewed and refined by OT faculty colleagues with expertise in research. Quantitative data and qualitative data were obtained from students using the questions highlighted in Table 1 and collected through a survey administered in Microsoft Teams.

**Table 1:** Pre-and post-assignment survey questions assessing confidence and perceptions of ChatGPT in intervention planning.

	Pre-survey	Post-survey
How confident are you in generating occupation-based interventions during your Level II fieldwork placements?	x	x
How confident are you in generating therapeutic exercise-based interventions during your Level II fieldwork placements?	x	x
How comfortable are you with using ChatGPT to generate intervention ideas for occupational therapy clinical practice?	x	x
How knowledgeable are you about ethical and safety considerations related to using AI technology like ChatGPT in occupational therapy practice?	x	x
How much do you believe ChatGPT technology can enhance treatment planning efficiency in occupational therapy practice?	x	x
To what extent do you believe ChatGPT can assist in generating diverse and relevant intervention ideas?		x
Do you think ChatGPT can contribute to improved efficiency in the intervention planning process?		x
Do you think evidence-based research findings should always align with suggestions generated by ChatGPT?		x
Describe the benefits of using ChatGPT to support intervention planning (Open Response)		x
Describe the limitations of using ChatGPT to support intervention planning (Open Response)		x

### Data analysis

Data analysis was performed using IBM SPSS version 28. Descriptive statistics were computed. Because the survey questions were measured on an ordinal scale, nonparametric tests were used. To determine whether there was a difference in responses before and after the intervention, Mann Whitney tests were performed. There was statistical significance if the *p*-value of a two-tailed test was ≤0.05.

The research team engaged in a qualitative analysis process to code narrative responses to questions and identify themes. The initial phase involved the primary researcher reviewing the narrative responses to the three questions individually, identifying initial themes or patterns, and coding the data accordingly.

Throughout the coding process, there was a continuous reflection on the emerging insights, the adequacy of the coding scheme, and the overall data analysis approach. This reflective practice led to refinements in the codes and themes, ensuring they accurately represented the students’ responses and aligned with the research objectives. The refined data and themes were then presented to an OT faculty researcher, who provided valuable insights and enhancements to the analysis. Subsequently, operational definitions for each theme were developed, allowing for a re-coding of the data by the primary researcher.

To enhance inter-rater reliability, these operational definitions were introduced to a graduate student who independently coded and sorted the data. This was followed by a collaborative session to revisit the coded data, ensuring that each response was accurately categorized within the agreed-upon themes. Discrepancies or uncertainties were addressed through dialog and consensus, fostering a rigorous and transparent analytical process. Despite these efforts to ensure inter-rater reliability, the coding process remains inherently subjective. Researchers’ interpretations may still influence the categorization of responses, which could affect the consistency of the findings.

Throughout this endeavor, the researchers documented their procedures and findings to maintain transparency and uphold the rigor of the qualitative analysis. This iterative approach facilitated the refinement and validation of themes, culminating in robust and trustworthy conclusions drawn from the narrative responses.

## Results

### Quantitative results

The study investigated OT students’ confidence in generating therapeutic interventions, their comfort with using ChatGPT for this purpose, their knowledge of ethical and safety considerations related to AI, and their perceptions of ChatGPT’s potential contributions to healthcare (See Table 2).

**Table 2:** Quantitative results of pre-and post-assignment survey data.

**Question**	**Time**	***n***	***M***	***SD***	***p***
How confident are you in generating occupation-based interventions that are during your Level II fieldwork placements?	Pre	34	3.15	0.78	0.066
	Post	27	3.52	0.70	
How confident are you in generating therapeutic exercise-based interventions during your Level II Fieldwork placements?	Pre	34	2.82	0.87	0.071
	Post	27	3.19	0.68	
How comfortable are you with using ChatGPT to generate intervention ideas for occupational therapy clinical practice?	Pre	34	2.03	1.09	<0.001
	Post	27	3.81	0.83	
How knowledgeable are you about ethical and safety considerations related to using AI technology like ChatGPT in occupational therapy practice?	Pre	34	2.32	1.09	<0.001
	Post	27	3.70	0.78	
How much do you believe ChatGPT technology can enhance treatment planning efficiency in occupational therapy practice?	Pre	34	3.24	1.02	0.005
	Post	27	3.89	0.70	
To what extent do you believe ChatGPT can assist in generating diverse and relevant intervention ideas?	Post	27	3.41	0.80	
				
On a scale from 1 to 5, how likely are you to recommend the integration of ChatGPT in occupational therapy education?	Post	27	3.63	0.93	

**Question**	**Time**	**Response**	**Count (%)**		
Do you think ChatGPT can contribute to improved efficiency in the intervention planning process?	Post	No	1 (3.7%)		
		Yes	26 (96.3%)		
Do you think evidence-based research findings should always align with suggestions generated by ChatGPT?	Post	No	13 (48.1%)		
		Yes	14 (51.9%)		

On a scale from 1 to 5, how likely are you to recommend the integration of ChatGPT in occupational therapy education?	Post	27	3.63	0.93								

**Question**	**Time**	**Response**	**Count (%)**			
Do you think ChatGPT can contribute to improved efficiency in the intervention planning process?	Post	No	1 (3.7%)			
		Yes	26 (96.3%)			
Do you think evidence-based research findings should always align with suggestions generated by ChatGPT?	Post	No	13 (48.1%)			
		Yes	14 (51.9%)			

#### Confidence in generating interventions

Students reported a slight, but not statistically significant, increase in confidence in generating occupational therapy intervention ideas (*p* = 0.07) and therapeutic exercise programs (p = 0.07) after exposure to ChatGPT.

#### Comfort with using ChatGPT

There was a significant increase in comfort with using ChatGPT to generate intervention ideas, with mean scores rising 88% from 2.03 (SD = 1.09) pre-exposure to 3.81 (SD = 0.83) post-exposure (*p* < 0.001).

#### Knowledge of ethical and safety considerations

Students also showed a significant improvement in their knowledge about ethical and safety considerations of using generative AI in healthcare settings, with mean scores increasing 59% from 2.32 (SD = 1.09) to 3.70 (SD = 0.78) (*p* < 0.001).

#### Perceived contributions of ChatGPT to healthcare

Students’ beliefs in ChatGPT’s potential to contribute to healthcare innovation and patient outcomes significantly increased 20% from a pre-exposure mean of 3.24 (SD = 1.02) to a post-exposure mean of 3.89 (SD = 0.70) (*p* = 0.005).

#### Anticipation of ChatGPT’s assistance

Post-exposure, students reported a belief that ChatGPT could assist in generating treatment plans, with a mean score of 3.41 (SD = 0.80).

#### Likelihood of future use

Post-exposure, students indicated a likelihood of using ChatGPT in their future practice, with a mean score of 3.63 (SD = 0.93).

#### Belief in improvement of patient outcomes

96.3% of students believed that ChatGPT could contribute to improved patient outcomes, while a split opinion was observed regarding whether evidence-based research findings should guide AI-generated intervention suggestions (Yes: 51.9%, No: 48.1%).

### Qualitative results

The qualitative analysis across three distinct questions related to the use of ChatGPT in occupational therapy intervention planning and fieldwork placements revealed insightful themes about its perceived benefits, limitations, and anticipated uses among occupational therapy students.

#### Time efficiency and idea generation

A significant portion of students highlighted ChatGPT’s role in reducing cognitive load and expediting the generation of treatment ideas, with 63% noting its efficiency and 85% appreciating its aid in creative ideation. These aspects are crucial in reducing the time for preliminary research and enhancing dynamic planning environments.

It quickly provides you with a long list of treatment ideas you can implement into practice.

ChatGPT can be used as helpful tool to help kick off the intervention planning process and help generate some ideas.

#### Adaptation and creativity

Challenges related to ChatGPT’s rigidity were noted by 11% of students, who felt it sometimes hindered personalized problem-solving, a key component in tailored client care.

Limits creativity of the therapist

The suggestions that ChatGPT provide are very general.

#### Evidence-based practice and client-centered care

Concerns were raised about the relevance and safety of ChatGPT-generated suggestions, with 36% of students skeptical of its evidence basis and 42% cautious of its lack of personalized client insights. This theme encapsulates the need for interventions to be both scientifically sound and tailored to individual client needs.

It is important to make sure the suggestions are supported by research.

ChatGPT does not know our patients personally like we do so they may suggest things we know won't work or be appropriate for the patient.

#### Safety and applicability

Echoing the need for cautious integration, 50% of students discussed the operational feasibility and the need for thorough vetting to ensure patient safety and relevance to specific conditions.

May not fully incorporate necessary precautions.

ChatGPT cannot always be trusted, because some suggestions could be dangerous or not good choices for a client.

#### Fieldwork and beyond

A vast majority (96%) indicated a strong intent to incorporate ChatGPT into their fieldwork, using it to foster initial treatment ideas and to alleviate cognitive load during planning stages. This anticipation extends to the belief in ChatGPT’s potential to assist in more general clinical tasks, noted by 7% of students, such as administrative work and research.

I think I will use it to generate ideas for my school-based placement since I don't have a lot of confidence in generating interventions for emotional regulation skills.

I will use ChatGPT to help write emails, summarize long articles, gather intervention ideas and more!

## Discussion

In the absence of specific research on OT students use of ChatGPT, this study highlights the potential of generative AI as a valuable tool in healthcare education, aligning with broader trends in AI adoption across various fields. While no prior studies have explored AI’s direct impact on OT intervention planning, the findings are consistent with research in related disciplines where AI has been shown to reduce cognitive load and improve clinical care (15). Similar increases in user comfort and acceptance of AI tools have been reported in medical and nursing education following exposure education or nursing (15, 16).

The significant improvement in students’ understanding of ethical and safety considerations mirrors concerns raised in the literature about responsible AI’s integration into healthcare (17). Divided opinions on evidence-based research aligning with AI-generated suggestions reflect ongoing discussions about balancing AI innovation with professional standards (9).

While this study is the first of its kind in OT education, the findings align with broader recognition of AI’s potential to streamline clinical processes and enhance creativity in intervention planning as a complement to traditional practices.

The findings underscore ChatGPT’s value in enhancing time efficiency and fostering creativity in intervention planning by accelerating the process and reducing cognitive burden. ChatGPT’s ability to inspire innovative, tailored intervention strategies highlights its role as a catalyst for creative thinking in clinical planning. These results emphasize the importance of incorporating technology like ChatGPT in education to foster effective, innovative clinical interventions.

This study has several limitations, including a small sample size, reliance on self-reported data, and a short-term intervention, which limit generalizability and long-term insights. While generative AI shows promise, concerns about the lack of evidence-based recommendations, safety issues, and the need for personalized care underscore the importance of teaching students to critically evaluate AI-generated suggestions. Though enthusiasm for the AI’s benefits is evident, careful management of its integration remains essential, emphasizing evidence-based practice and professional expertise.

## Conclusion

The findings of this novel study suggest a positive disposition toward integrating ChatGPT into occupational therapy education, driven by its potential to enhance creative ideation, time efficiency, and personalized care. Future research should focus on the broader implications of integrating generative AI into health professions education, exploring its role in improving student outcomes during clinical placements, and developing robust educational frameworks to equip both students and practitioners with the skills needed to effectively integrate AI tools into their practice. The study underscores the necessity of a careful and informed approach to the integration of AI in clinical education, highlighting the potential for ChatGPT and similar technologies to augment, rather than replace, the critical reasoning and expertise of practitioners.

## Data Availability

The original contributions presented in the study are included in the article/supplementary material, further inquiries can be directed to the corresponding authors.
